# Effects of exposure to environmentally relevant concentrations of lead (Pb) on expression of stress and immune-related genes, and microRNAs in shorthorn sculpins (*Myoxocephalus scorpius*)

**DOI:** 10.1007/s10646-022-02575-x

**Published:** 2022-08-25

**Authors:** Khattapan Jantawongsri, Rasmus Dyrmose Nørregaard, Lis Bach, Rune Dietz, Christian Sonne, Kasper Jørgensen, Syverin Lierhagen, Tomasz Maciej Ciesielski, Bjørn Munro Jenssen, Courtney Alice Waugh, Ruth Eriksen, Barbara Nowak, Kelli Anderson

**Affiliations:** 1 Institute for Marine and Antarctic Studies (IMAS), University of Tasmania, Launceston, TAS 7250 Australia; 2 Department of Ecoscience and Arctic Research Centre (ARC), Faculty of Technical Sciences, Aarhus University, Frederiksborgvej 399, P.O. Box 358, DK-4000 Roskilde, Denmark; 3 Den Blå Planet, National Aquarium Denmark, Jacob Fortlingsvej 1, DK-2770 Kastrup, Copenhagen, Denmark; 4 Department of Chemistry, Norwegian University of Science and Technology, NO-7491 Trondheim, Norway; 5 Department of Biology, Norwegian University of Science and Technology, Høgskoleringen 5, NO-7491 Trondheim, Norway; 6 Department of Arctic Technology, The University Centre in Svalbard, P.O. Box 156, NO-9171 Longyearbyen, Svalbard, Norway; 7 Faculty of Biosciences and Aquaculture, Nord University, NO-7729 Steinkjer, Norway; 8 CSIRO Oceans and Atmosphere, Castray Esplanade, Battery Point, Hobart, TAS 7004 Australia

**Keywords:** Arctic lead–zinc mines, Dissolved Pb exposure, Gene expression, Greenland sculpin, Immune-related gene, Metal stress-related gene

## Abstract

Old lead–zinc (Pb–Zn) mining sites in Greenland have increased the environmental concentration of Pb in local marine organisms, including the shorthorn sculpin. Organ metal concentrations and histopathology have been used in environmental monitoring programs to evaluate metal exposure and subsequent effects in shorthorn sculpins. So far, no study has reported the impact of heavy metals on gene expression involved in metal-related stress and immune responses in sculpins. The aim of this study was to investigate the effect of exposure to environmentally relevant waterborne Pb (0.73 ± 0.35 μg/L) on hepatic gene expression of *metallothionein* (*mt*), *immunoglobulin M* (*igm*), and microRNAs (miRNAs; *mir132* and *mir155*) associated with immune responses in the shorthorn sculpin compared to a control group. The *mt* and *igm* expression were upregulated in the Pb-exposed group compared to the control group. The transcripts of *mir132* and *mir155* were not different in sculpins between the Pb-exposed and control group; however, miRNA levels were significantly correlated with Pb liver concentrations. Furthermore, there was a positive correlation between liver Pb concentrations and *igm*, and a positive relationship between *igm* and *mir155*. The results indicate that exposure to Pb similar to those concentrations reported in in marine waters around Greenland Pb–Zn mine sites influences the *mt* and immune responses in shorthorn sculpins. This is the first study to identify candidate molecular markers in the shorthorn sculpins exposed to waterborne environmentally relevant Pb suggesting *mt* and *igm* as potential molecular markers of exposure to be applied in future assessments of the marine environment near Arctic mining sites.

## Highlights

- *mt* and *igm* were upregulated in Pb exposed fish compared to the control group.
- Significant positive correlation was found between Pb concentrations in liver and *igm*.
- Expression of *igm* was positively correlated to *mir155* expression.
- *mir155* expression was positively correlated with severity scores of hepatic lesions.
- Pb levels in the liver were significantly correlated with miRNAs expression.

## Introduction

Heavy metal pollution from industrial, mining, and agricultural sources in aquatic ecosystems has been of concern across the world, including the Arctic (Dietz et al. 1998; Evans et al. 2000; Voigt 2003; Chua et al. 2018). Lead (Pb) is a non-essential and toxic heavy metal that is widespread in aquatic environments (Scheuhammer et al. 2008). Exposure to Pb even at low concentrations impairs biological functions, such as reproduction, development, behavior, learning, immune response, and metabolism (Eisler 1988). Pb is particularly harmful to aquatic organisms, including fish, as it bioaccumulates through uptake via gills, dietary consumption, and contaminated sediments (Eisler 1988; Scheuhammer et al. 2008; Mager 2011). In fish, Pb accumulates in liver, spleen, and kidney, as well as the digestive system and gills (Jezierska and Witeska 2006). A wide range of Pb concentrations (1 to 5.15 mg/L) have been demonstrated to activate oxidative stress and cause inflammation in different fish species (Lee et al. 2019; Jing et al. 2020).

Metallothioneins (Mt) are low molecular weight metal binding proteins involved in homeostatic regulation and transportation of essential metals, such as copper (Cu) and zinc (Zn) (Hogstrand and Haux 1990; Coyle et al. 2002; Baird et al. 2006). Mt proteins are also involved in detoxification of non-essential metals, including Pb, to protect tissues from oxidative stress (Hogstrand and Haux 1990; Dallinger et al. 1997; Monteiro et al. 2011). Production of Mt is induced by exposure to heavy metals and Mt is measured to estimate the stress responses to heavy metal exposure, such as Pb, across fish species (Schmitt et al. 2007; Huang et al. 2014; Yin et al. 2018b). Following exposure to varying Pb concentrations (0.07 to 1.16 mg/L), the expression of *mt* increased and was suggested as biomarker of exposure to Pb (Huang et al. 2014). Expression of *mt* has been applied as a sensitive biomarker of metal exposure in fish in metal-contaminated environments (Cheung et al. 2004). For example, Wang et al. (2014) showed that expression of *mt* in the rare minnow (*Gobiocypris rarus*) was upregulated possibly as a result of heavy metal exposure and oxidative stress.

Heat shock proteins (Hsp), also known as “stress proteins”, are involved in a variety of physiological activities, including protein chaperoning, apoptosis protection, steroidogenesis, and stress resistance (Mahmood et al. 2014). Exposure to heavy metals leads to numerous cellular heat-shock responses, including induction of Hsp to protect cellular functions (Sanders 1993). Hsp, such as Hsp70, are highly conserved proteins in fish and are applied as a potential biomarker to assess cellular stress responses in fish exposed to heavy metals, including Pb (Basu et al. 2002; Kim and Kang 2016). In addition, the expression of *hsp* can be influenced by a number of factors, including heat and cold shock, xenobiotics, and pathogens (Iwama et al. 1998; Lewis et al. 1999; Basu et al. 2002). Previous studies have shown that the expression of *hsp70* in fish was elevated following exposure to heavy metals, including various Pb concentrations (0.05–800 mg/L waterborne Pb) (Yin et al. 2018b; Zhao et al. 2020).

Exposure to Pb alters immune response and induces immunomodulation in fish (Zelikoff 1993; Zelikoff et al. 1995; Luebke et al. 1997; Bols et al. 2001; Qian et al. 2020). Immunoglobulin M (IgM) is the most highly conserved and abundant immunoglobulin isotype in teleosts and is one of the most essential components of the immune system as it mediates humoral adaptive immunity in fish to eliminate invading pathogens (Salinas et al. 2011; Zwollo 2018; Smith et al. 2019). IgM has been used as an indicator of immune response in teleosts (Wester et al. 1994; Lee et al. 2014). Previous studies of rockfish (*Sebastes schlegelii*) demonstrated that dietary exposure to Pb activated an immune response, increasing plasma IgM concentration (Kim and Kang 2016). In contrast, Zhao et al. (2020) showed that waterborne Pb exposure decreased serum IgM concentration in the northern snakehead (*Channa argus*). MicroRNAs (miRNAs) are important regulators of the immune response and expression of immune associated miRNAs can be modulated in many different species by exposure to environmental pollutants (O’Connell et al. 2007; Mehta and Baltimore 2016; Andreassen and Høyheim 2017; Li et al. 2019; Badry et al. 2020; Sun et al. 2021). Recent research found that miRNAs, such as *mir132* and *mir155*, play critical roles in regulating inflammation, suggesting they are crucial regulators of immune responses (Rodriguez et al. 2007; Roy and Sen 2010; He et al. 2014; Ma et al. 2018; Zhao et al. 2022). There is some evidence that the alteration of *mir155* expression could be a novel biomarker of exposure to pollution (Huang et al. 2016; Badry et al. 2020). For example, *mir155* was downregulated in adult zebrafish (*Danio rerio*) after exposure to an insecticide fipronil (Huang et al. 2016). In addition, miRNAs, including *mir132*, have been identified as important miRNAs associated with responses to exposure to metals (Pellegrini et al. 2016).

Although previous studies have proposed molecular markers for assessing the effects of exposure to metals on stress and immune responses in many fish species, the study of molecular endpoints to identify candidate molecular markers remains a knowledge gap in benthic species, including the sculpins.

Previous field studies on the impact of metal pollution at historic Pb–Zn mining sites in Greenland used shorthorn sculpins (*Myoxocephalus scorpius*) as a sentinel species to assess aqueous exposure and effects of toxic elements, including Pb, on bioaccumulation (e.g., resulting in Pb residues of 0.01–0.94 μg/g in liver and 0.01–0.69 μg/g in muscle) and histology (Sonne et al. 2014; Dang et al. 2017, 2019; Nørregaard et al. 2018; Hansson et al. 2020). *M. scorpius* is a relatively sedentary and benthic marine fish species that lives in the North Atlantic coast and the Arctic Ocean (Luksenburg and Pedersen 2002; Thorsteinson and Love 2016). In Greenland, *M. scorpius* is abundant at both western and eastern Greenland mine sites, and easy to catch by angling near mine sites (Søndergaard and Mosbech 2022). Recently, the effects of Pb exposure on shorthorn sculpin, under controlled laboratory conditions, have corroborated field observations, including bioaccumulation in organs and blood, and histopathology of liver and gills (Jantawongsri et al. 2021). However, there has been no research on the effects of exposure to Pb on stress and immune responses in shorthorn sculpin, or any other species in this genus. Thus, our aim was to investigate the expression of stress-related and immune-related genes in shorthorn sculpins exposed to Pb concentrations that are relevant for the marine environment adjacent to Greenland Pb–Zn mines. Following a controlled laboratory experiment, hepatic expression of *mt*, *igm*, *hsp70*, and miRNAs were investigated in control and Pb-exposed fish. The aim was to assess the potential of these stress and immune-related genes as molecular markers of Pb exposure in sculpins around Pb–Zn mines in the Arctic, including Greenland.

## Methods

### Experimental design

For a detailed description of the experiment, see Jantawongsri et al. (2021). Briefly, wild-caught sculpins (15 fish in each of two Pb-exposed tanks and 15 fish in each of two control tanks) were exposed to an environmentally-relevant concentration of dissolved Pb (0.73 ± 0.35 μg/L (mean ± standard deviation, SD)) consistent with a previous report on seawater near the former Black Angel Pb–Zn mine in Maarmorilik, West Greenland (0.46 μg/L of dissolved Pb; Søndergaard et al. 2011). At the end of the experiment, a liver sample was collected from each fish and fixed in RNAlater (Ambion, Austin, TX, USA), incubated at 4 °C overnight and then stored at −20 °C. As there were no significant variations in biometrics, age, or residues of other elements (excluding Pb concentrations) between control and exposed sculpins caught in the same area, it was assumed there was no background difference between the fish before the experiment (Jantawongsri et al. 2021). After 28 days of exposure, liver residues of Pb were significantly higher in Pb-exposed sculpins (0.50 ± 0.23 μg/g dry weight) than in control fish (0.13 ± 0.10 μg/g dry weight) (*p* < 0.001; Jantawongsri et al. 2021).

### RNA isolation and cDNA synthesis

RNA extraction and cDNA synthesis were performed on 22 control sculpins and 20 Pb-exposed sculpins following the method of Castaño-Ortiz et al. (2019). RNA was extracted from the liver samples (approx. 50 mg) using the miRNeasy Mini Kit (Qiagen, Oslo, Norway) following the manufacturer’s protocol and stored at −20 °C. RNA concentration was then determined using a NanoDrop^®^ ND-2000cUV-visible Spectrophotometer (NanoDrop Technologies, Wilmington, USA). cDNA synthesis was performed using 500 ng of RNA and the miRCURY LNA™ RT Kit (Qiagen, Oslo, Norway) as per manufacturer’s instructions.

### Partial isolation of candidate genes and qPCR primer design

Target genes in this study represented (1) metal-ion binding protein (*mt*), (2) immune-related (*igm*), (3) heat shock protein (*hsp70*), and (4-5) miRNAs associated with immune response (*mir132* and *mir155*) (Table 1). To amplify fragments of *mt*, *igm*, and *hsp70* gene from *M. scorpius*, the mRNA nucleotide sequences from shorthorn sculpin-related species retrieved from GenBank^®^ database (NCBI) were aligned using the Clustal Omega multiple sequence alignment tool (https://www.ebi.ac.uk/Tools/msa/clustalo/) and degenerate oligonucleotide primers were designed from the conserved regions (Pankhurst et al. 2011) (Table S1). *mir132* and *mir155* primers were commercially designed by miRCURY LNA™ miRNA PCR Assays (Qiagen, Oslo, Norway).

**Table 1:** qPCR primers

Gene	Primer sequence (5’ → 3’)	Amplicon size (bp)	Tm (°C)	NCBI/miRBase accession number	E
*hsp70*	F: GGT GTC CAA CGC AGT CAT C	119	64.4	OK668366	1.86
	R: CCG TCG GCT CGT TGA TGA T		65.1		
*igm*	F: TAT TTC GTG GGA GAA CCA GG	178	63.7	OK668365	1.92
	R: GGG TGT CTT AAG TGG TAC CAT CC		64.3		
*mt*	F: GAG GAT CCT GCA CCT GCA A	124	66.6	OK668364	1.95
	R: GTG TCG CAC GTC TTC CCT TT		65.4		
*mir132*	*ola-miR-132* (5’ UAA CAG UCU ACA GCC AUG G) amplified by miRCURY LNA™ miRNA PCR Assays, catalog number: YP02103600 (Qiagen, Oslo, Norway)			MIMAT0022617 (Li et al. 2010)	1.92
*mir155*	*dre-miR-155* (5’ UUA AUG CUA AUC GUG AUA GGG G) amplified by miRCURY LNA™ miRNA PCR Assays, catalog number: YP02102917 (Qiagen, Oslo, Norway)			MIMAT0001851 (Chen et al. 2005)	1.72

*bp* base pairs, *Tm* melting temperature, *E* efficiency

PCR amplification for *mt*, *igm*, and *hsp70* was carried out using Taq PCR Core Kit (Qiagen, VIC, Australia) according to the manufacturer’s specifications with 10 μM of each primer. Amplification was performed on Bio-Rad C1000™ thermal cycler using the following cycling conditions: 3 min at 94 °C, then 40 cycles of 94 °C for 30 s, 56 °C (*mt*) or 57 °C (*igm*) or 52 °C (*hsp70*) for 30 s and 72 °C for 30 s, followed by a final extension at 72 °C for 10 min.

PCR products were separated via gel electrophoresis in 2% agarose gel and purified from the gel by using ISOLATE II PCR and Gel Kit (Bioline, NSW, Australia). Purified PCR products were quantified using Qubit^®^ dsDNA BR Assay Kits (Thermo Fisher Scientific, VIC, Australia) then sent to Griffith University DNA Sequencing Facility (GUDSF; Griffith University, Nathan, QLD, Australia) for Sanger sequencing. Sequencing data was assessed using Chromas Version 2.6.6 (Technelysium, QLD, Australia) and sequences were submitted to GenBank. These sequences were used to design qPCR primers (Primer-BLAST, https://www.ncbi.nlm.nih.gov/tools/primer-blast/) to have a melting temperature between 63–67 °C and produce an amplicon between 100–200 bp (Table 1).

### qPCR procedure

Each qPCR reaction for *mt*, *igm*, and *hsp70* contained: 5 μL SsoAdvanced™ Universal SYBR^®^ Green Supermix (Bio-Rad, NSW, Australia), 100 nM each primer, 4 ng of cDNA template, and water to a final volume of 10 μL. Each *mir132* and *mir155* reaction contained: 5 μL miRCURY LNA™ SYBR^®^ Green PCR Kits (Qiagen, Oslo, Norway), 10 μM each primer, 2.5 ng of cDNA template, and water to a final volume of 10 μL. All qPCRs were performed in duplicate.

qPCRs for *mt*, *igm*, and *hsp70* were performed on CFX96™ real-time PCR detection system (Bio-Rad, NSW, Australia). A touch-down qPCR protocol was used according to the guidelines of Zhang et al. (2015) as follows: one cycle 95 °C for 3 min and four cycles 95 °C for 20 s, 66 °C for 10 s by decreasing 3 °C per cycle, followed by 40 cycles of 95 °C for 15 s, 58–60 °C for 15 s. All primers were evaluated for specificity at the end of cycle 40 using melt curve analysis, which comprised of a 1 °C per 5 s temperature gradient from 60–94 °C. qPCRs for *mir132* and *mir155* were conducted on Roche LightCycler^®^ 96 (Roche Diagnostics, Basel, Switzerland) with the following running conditions: 2 min at 95 °C, two steps cycling at 10 s at 95 °C and 60 s at 56 °C for 40 cycles followed by a melt curve (as per Table S2). Duplicate no template controls (NTCs) were used in each qPCR plate and no contamination was detected.

The efficiency of each individual sample was calculated from the slopes of amplification curves and averaged for each gene using a window-of-linearity approach in LinRegPCR software (version 2020.2) (Ramakers et al. 2003; Ruijter et al. 2009). qPCR primers were considered acceptable based on the following criteria: (1) the estimated efficiency was between 1.7 and 2.0 (Wilkerson et al. 2013; Kim et al. 2017b), (2) the melting curve presented one single peak, and (3) no primer-dimers formed in reactions containing template (Rodríguez et al. 2015).

### Data analysis

A Bayesian Markov Chain Monte Carlo (MCMC) chain algorithm was conducted to evaluate the response of target mRNA/miRNA to experimental factors. The unit of biological replication used was an individual fish, so the replication level was *n* = 9–11 per tank and *n* = 20–22 per treatment. Hepatic gene expression levels were determined using a reference gene-free approach and the MCMC.qpcr package, implemented in *R* Version 3.6.1 (R Core Team 2021) following the procedures proposed by Matz et al. (2013). In MCMC, a two-way design “naïve” model was fitted to estimate the expression of target genes in response to fixed effects of “treatment” (control and Pb exposure) and “tank” (2 control and 2 Pb exposure tanks), and random effect (sample) as follows:

$$\begin{array}{ll}{{{\mathrm{ln}}}}\left( {{{{\mathrm{rate}}}}} \right) \sim {{{\mathrm{gene}}}} + {{{\mathrm{gene}}}}:{{{\mathrm{Treatment}}}}\\ \qquad\qquad+ {{{\mathrm{gene}}}}:{{{\mathrm{Tank}}}}:{{{\mathrm{Treatment}}}} + \left[ {{{{\mathrm{sample}}}}} \right]\end{array}$$

Gene expression data were reported as log2 transcript abundances in posterior mean (model estimates) with 95% credible intervals (CIs). The credible intervals are the Bayesian analog of confidence intervals. The statistical significance of changes in expression were evaluated using MCMC, with a significance threshold of *p* < 0.05 (Matz et al. 2013).

Transcript abundances (normalized data) from individual sculpin for all target genes were used to analyze the relationship between gene expression (this study) and data previously reported by Jantawongsri et al. (2021); i.e., body mass, length, liver mass, condition factor, hepato-somatic index (HSI), age, histology (including severity score of lesions in liver and gills, number of digeneans parasites in gills, number of mucous cells/interlamellar unit (ILU) in gills), and concentrations of Pb in liver, gills, muscle, and blood. For a detailed description of the histology and metal analyses, see Jantawongsri et al. (2021). Spearman’s rank correlation was then analyzed using the Hmisc package (Harrell 2015) and stats package in *R* (R Core Team 2021), and correlation coefficients (*r*_s_) with *p* < 0.05 were considered significant.

## Results

### Hepatic gene expression

**Fig. 1:**
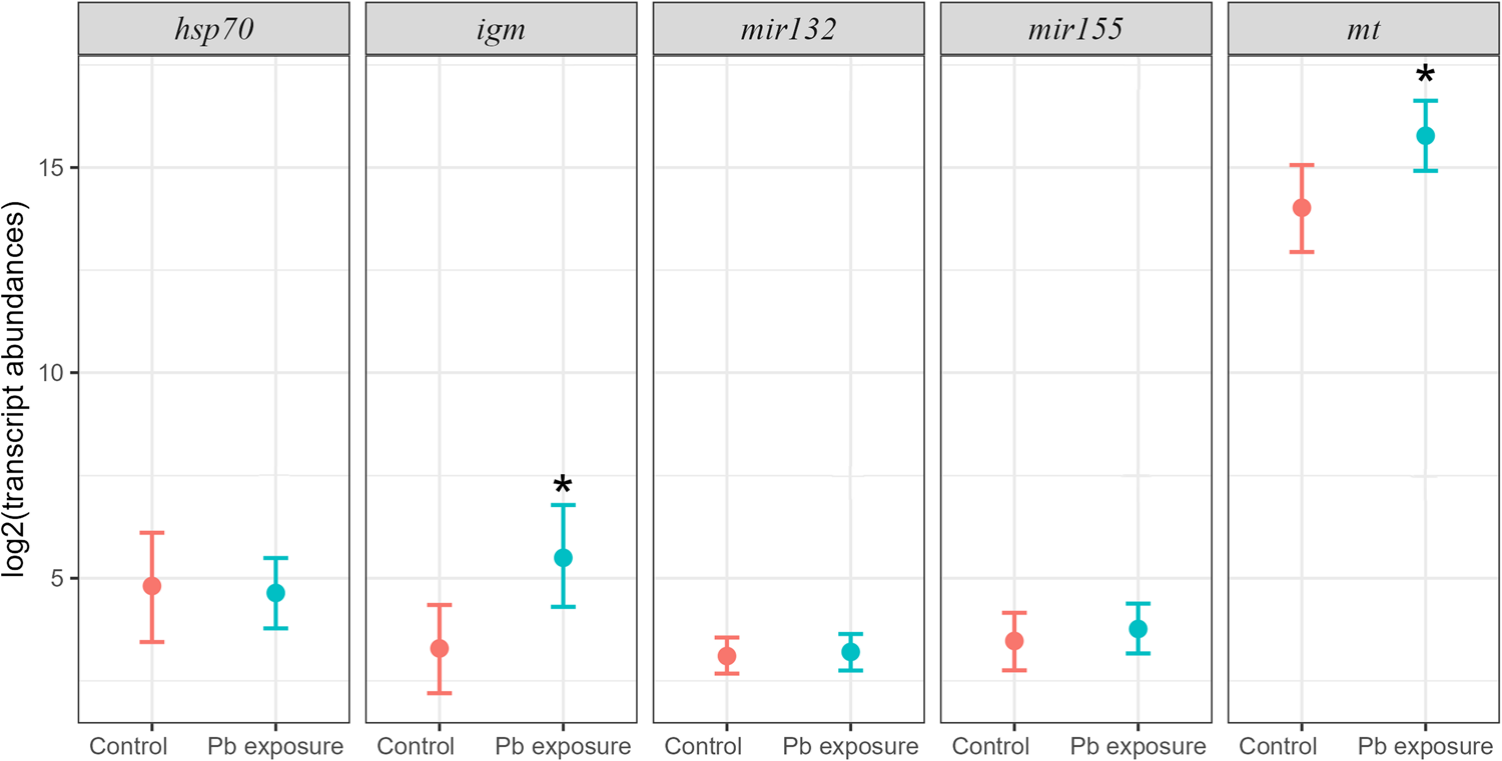
Hepatic gene expression (model-derived log2 transcript abundance) for *hsp70*, *igm*, *mir132*, *mir155* and *mt* in control and Pb-exposed sculpins (*M. scorpius*). Data represent mean ± 95% CIs of the posterior distribution (*n* = 22 for control and *n* = 20 for Pb exposure). Asterisk (*) indicates statistically significant difference between experimental groups for each gene (*p* < 0.05)

Hepatic expression levels of *mt* mRNA were significantly greater in the Pb-exposed sculpins compared to those control sculpins (1.24-fold change, *p* = 0.030; Fig. 1 and Table S3). Similarly, *igm* mRNA levels were significantly higher in Pb-exposed fish than in control fish (1.53-fold change, *p* = 0.028; Fig. 1 and Table S3). In contrast, *hsp70* mRNA levels and the transcripts of *mir132* and *mir155* did not differ significantly between the Pb-exposed and control sculpins (*p* > 0.05; Fig. 1 and Table S3). The replicate tanks were pooled to compare Pb-exposed and control sculpin as no significant differences were observed in hepatic mRNA levels of *mt*, *igm*, and *hsp70*, and transcripts levels of *mir132* and *mir155* in the sculpins among the tanks (*p* > 0.25; Table S3).

### Relationships between gene expression and other parameters

**Fig. 2:**
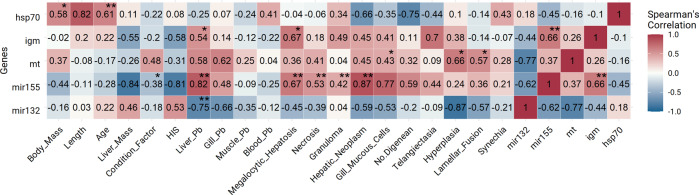
Spearman’s rank correlation coefficients, *r*_s_ (−1 ≥ *r*_s_ ≥ +1), between gene expressions (current study) and data published in Jantawongsri et al. (2021), i.e., biometrics, Pb concentrations in organs and blood, and histology (severity of lesion in organs, gill mucous cells, and parasites) of the shorthorn sculpins exposed to Pb for 28 days, with ‘*’ indicating statistically significant correlations at *p* < 0.05 and ‘**’ at *p* < 0.01

Transcript levels of *hsp70* of all sculpins were positively correlated with body mass (*r*_s_ = 0.58, *p* = 0.019, *n* = 23; Fig. 2) and age (*r*_s_ = 0.61, *p* = 0.004, *n* = 21; Fig. 2). A significant positive correlation was observed between *igm* and *mir155* expression in all sculpins (*r*_s_ = 0.66, *p* = 0.001, *n* = 39; Fig. 2). Expressions of *igm* (*r*_s_ = 0.54, *p* = 0.038, *n* = 20; Fig. 2) and *mir155* (*r*_s_ = 0.82, *p* = 0.008, *n* = 22; Fig. 2) were positively correlated with hepatic Pb concentrations of all sculpins. There were statistically significant positive correlations between expression of *mt* and number of mucous cells/ILU in the gills (*r*_s_ = 0.43, *p* = 0.036, *n* = 42; Fig. 2) and severity score of gill lesions, including: hyperplasia (*r*_s_ = 0.66, *p* = 0.012, *n* = 42; Fig. 2) and complete lamellar fusion (*r*_s_ = 0.57, *p* = 0.014, *n* = 42; Fig. 2). Moreover, transcript of *mir155* of all fish was positively correlated with severity scores of hepatic lesions, including megalocytic hepatosis (*r*_s_ = 0.67, *p* = 0.002, *n* = 42; Fig. 2), necrosis (*r*_s_ = 0.53, *p* < 0.001, *n* = 42; Fig. 2), granuloma (*r*_s_ = 0.42, *p* = 0.007, *n* = 42; Fig. 2) and hepatic neoplasm (*r*_s_ = 0.87, *p* = 0.001, *n* = 42; Fig. 2), but negatively corelated with condition factor (*r*_s_ = −0.8738, *p* = 0.02, *n* = 41; Fig. 2). There was significant negative correlation between expression of *mir132* and Pb concentrations in liver (*r*_s_ = −0.75, *p* = 0.008, *n* = 22; Fig. 2).

## Discussion

In teleosts, Mt induction, and hence increased expression of *mt*, could occur in response to oxidative stress caused by exposure to heavy metals such as Cu, cadmium (Cd), mercury (Hg), nickel (Ni), Pb, and Zn (Schlenk et al. 1999; Cheung et al. 2004; Tom et al. 2004; Schmitt et al. 2007). In the present study, significant up-regulation of hepatic *mt* was observed in the liver of shorthorn sculpins exposed to Pb for 28 days compared to the unexposed control sculpins. This finding is consistent with results from other species (Man and Woo 2008; Rhee et al. 2009). An induction of *mt* mRNA was observed in the liver of tilapia following 10 mg/kg of Pb intraperitoneal injection (Cheung et al. 2004). Dietary Pb exposure resulted in a significant increase in hepatic *mt* expression in juvenile Korean rockfish, *S. schlegelii* (Kim et al. 2017a). In our study, *mt* transcripts correlated significantly with the number of mucous cells/ILU in the gills and the severity score of gill lesions, including hyperplasia and complete lamellar fusion. Collectively, this suggests that exposure to Pb, incorporates a variety of responses, including increase in the number of mucous cells, histological alterations, and metal-related stress gene transcription (Hansen et al. 2007; Fu et al. 2017; Abril et al. 2018).

Exposure to heavy metals, such as Cd, Hg, and Pb, can affect the fish immune response (Zelikoff 1993; Zelikoff et al. 1995; Yu et al. 2020). A previous study on liver transcriptome of juvenile largemouth bass (*Micropterus salmoides*) revealed that acute exposure (96 h) to Pb (0, 10, 17.8, 31.6, 56.2, and 100 mg/L) activated various pathways related to the immune response, including complement pathway, coagulation cascades, antigen processing and presentation, and natural killer cell-mediated cytotoxicity (Qian et al. 2020). Yin et al. (2018b) reported an increase in *igm* mRNA expression in gibel carp (*Carassius auratus gibelio*) exposed to Pb. Likewise, our study showed that hepatic *igm* expression was significantly upregulated in the Pb-exposed shorthorn sculpins compared to the control sculpins. Furthermore, when pooling both control fish and exposed fish, the expression of the *igm* was positively correlated with liver Pb. This suggests that the upregulated hepatic *igm* transcripts in sculpins were associated with exposure to environmentally relevant Pb concentrations, which may result in functional alterations of humoral mechanisms (e.g., antibody production) of the immune response.

Hsp, such as Hsp70, are involved in a variety of physiological processes and play a vital role in homeostasis of proteins and cellular stress responses in fish (Sanders 1993; Iwama et al. 1999; Basu et al. 2003; Yin et al. 2018a). In teleosts, *hsp* genes have been differentially expressed due to different stressors, such as, dose-dependent synergistic effects of toxicants and other environmental factors (Basu et al. 2002; Eichler et al. 2005; Tine et al. 2010; Mahmood et al. 2014). For example, the expression of *hsp70* in liver and gill of *C. argus* increased after waterborne Pb exposure (50, 200, and 800 mg/L Pb) for 14 and 28 days (Zhao et al. 2020). Following exposure to Pb (0.05, 0.5, and 1 mg/L) for 60 days *hsp70* was upregulated in the spleen of *C. gibelio* (Yin et al. 2018b). In this study, however, hepatic *hsp70* of shorthorn sculpins was not significantly different between the control and Pb exposed sculpins. The lack of induction of *hsp70* of the sculpins may be due to the exposure time and the lower Pb concentration relative to other studies (Ribecco et al. 2011; Yin et al. 2018b; Zhao et al. 2020).

Changes in the expression of miRNAs are known to be involved in the regulation of genes related to metabolism, apoptosis, and immune-related signaling pathways in fish following toxicant exposure, stressors, or diseased states (Chen 2010; Kure et al. 2013; Gao et al. 2014; Ahkin Chin Tai and Freeman 2020; Balasubramanian et al. 2020). Previous studies have shown that *mir155* plays an important function in inflammation (Badry et al. 2020; Jing et al. 2020). For example, Ma et al. (2019) reported that exposure to 1-methyl-3-octylimidazolium bromide ([C_8_mim]Br) upregulated *mir155* on silver carp (*Hypophthalmichthys molitrix*), suggesting that this miRNA may be involved in the inflammatory response in fish. In this study, transcripts of *mir155* were positively correlated with *igm* transcripts as well as hepatic Pb concentrations, severity scores of hepatic lesions (i.e., megalocytic hepatosis, necrosis, granuloma, and hepatic neoplasm). The expression of *mir155* was positively correlated with the mRNA levels of proinflammatory cytokines, including tumor necrosis factor alpha (TNF-α) in head kidney of Asian carp (Jing et al. 2020). Expression of *mir132* was also found to be negatively associated with liver Pb levels in this study. However, there are few reports on how *mir132* regulates fish immune responses. A previous study on miiuy croaker (*Miichthys miiuy*) showed that *mir132* is a negative regulator of fish inflammatory cytokine production implicated in the immune response induced by lipopolysaccharides (LPS) (Dong et al. 2021). Further research to determine the specific target genes of miRNAs and their function related to the immune response particularly in sculpin with regards to heavy metals exposure is necessary to understand the underlying regulatory processes of miRNA expression.

## Conclusions

In conclusion, the present study evaluated the potential toxicity of Pb exposure on gene expression associated with stress (*mt* and *hsp70*) and immune response (*igm*, *mir132*, and *mir155*) in the shorthorn sculpin, *M. scorpius*. The results demonstrated that exposure of shorthorn sculpin to environmentally relevant dissolved Pb concentration induced an increase in hepatic *mt* and *igm* expression. Expression of *igm* was positively correlated to Pb concentration in the liver. There were positive correlations between *mir155* and *igm* and hepatic Pb concentration in liver, while *mir132* was negatively correlated with Pb. Prior to this study, there was no information on effect of metal exposure on gene expression in marine sculpin. This study was the first to report that Pb exposure can affect expressions of hepatic metal homeostasis and immune response-related genes in the shorthorn sculpin. Overall, our results suggest that up-regulation of hepatic *mt* and *igm* has a potential as a biomarker of exposure to Pb which could improve the assessment of impacts of mining in the Arctic, including Greenland. However, further research is needed to evaluate their applications.

## Supplementary Information

Supplementary Information

## Data Availability

Data are available from the corresponding author.
