# Supplementary material for: Effects of exposure to environmentally relevant concentrations of lead (Pb) on expression of stress and immune-related genes, and microRNAs in shorthorn sculpins (Myoxocephalus scorpius)
Source: Ecotoxicology. 2022 Aug 25;31(7):1068–77. doi: 10.1007/s10646-022-02575-x (PMC9458575; doi:10.1007/s10646-022-02575-x)
Supplement: Supplementary file 1 — Supplementary Information [file 10646_2022_2575_MOESM1_ESM.pdf]

Supplementary Information

Effects of exposure to environmentally relevant concentrations of lead (Pb) on expression of stress and immune-related genes, and microRNAs in shorthorn sculpins (*Myoxocephalus scorpius*)

Khattapan Jantawongsri[a,*], Rasmus Dyrmose Nørregaard[b], Lis Bach[b], Rune Dietz[b], Christian Sonne[b], Kasper Jørgensen[c], Syverin Lierhagen[d], Tomasz Maciej Ciesielski[e], Bjørn Munro Jenssen[b,e,f], Courtney Alice Waugh[e,g], Ruth Eriksen[a,h], Barbara Nowak[a,b], Kelli Anderson[a]

a. Institute for Marine and Antarctic Studies (IMAS), University of Tasmania, Launceston, Tasmania 7250, Australia
b. Department of Ecoscience and Arctic Research Centre (ARC), Faculty of Technical Sciences, Aarhus University, Frederiksborgvej 399, P.O. Box 358, DK-4000, Roskilde, Denmark
c. Den Blå Planet, National Aquarium Denmark, Jacob Fortlingsvej 1, DK-2770 Kastrup, Copenhagen, Denmark
d. Department of Chemistry, Norwegian University of Science and Technology, NO-7491, Trondheim, Norway
e. Department of Biology, Norwegian University of Science and Technology, Høgskoleringen 5, NO-7491, Trondheim, Norway
f. Department of Arctic Technology, The University Centre in Svalbard, P.O. Box 156, NO-9171 Longyearbyen, Svalbard, Norway
g. Faculty of Biosciences and Aquaculture, Nord University, NO-7729, Steinkjer, Norway
h. CSIRO Oceans and Atmosphere, Castray Esplanade, Battery Point, Hobart, Tasmania 7004, Australia

* **Corresponding Author:**
Khattapan Jantawongsri
Affiliation: Institute for Marine and Antarctic Studies (IMAS), University of Tasmania, Launceston, Tasmania 7250, Australia
Phone: +61 431647481
Email: khattapan.jantawongsri@utas.edu.au

Numbers of pages: 5
Numbers of tables: 3
Prepared for short communication, journal *Ecotoxicology*, 2022

Table S1 List of fish species and sequence IDs for degenerate primer design

**Gene**	**Fish species and sequence IDs used to design primers**	**Primer sequence (5'→3')**	**Ta (°C)**	**Product size (bp)**
mt	*Cottus gobio* (MN577630.1)	F: CTC CAA GA**S** TGG **M**AC CTG C	56	256
	Cyclopterus lumpus (XM_034535227.1)	R: GTT **R**TC TGT **W**GA CAT TTG CAA		
	Thermarces Cerberus (X97277.1)			
	Zoarces viviparus (X97270.1)			
igm	*Bovichtus diacanthus* (EU884292.1)	F: ACT GTA CAG **Y**GC AGC AAG TT	57	592
	Dissostichus mawsoni (EU488726.1)	R: ATC ATC AAC **K**AG CCA AG**K** CAC A		
	Epinephelus akaara (HQ007252.2)			
	Pagetopsis macropterus (EU195861.1)			
hsp70	*Oligocottus maculosus* (DQ013308.1, DQ013309.1)	F: TGA ACC CCA **R**CA ACA CTG T	52	420
		R: CC**Y** AGG TC**Y** AAG AT**S** AGG ACG		

mt = metallothionein, *igm = immunoglobulin M*, *hsp70 = heat shock protein 70*; Ta = annealing temperature; bp = base pairs; degenerate bases are: R = A,G; Y = C,T; M = A,C; K = G,T; S =C,G; W = A,T

Table S2 qPCR cycling conditions

Gene	Cycle function	No. cycles	Temperature (°C)	Time
hsp70	Initial denaturation	1	95	3 min
	Denaturation<br>Touchdown stage	4	95<br>66 (by decreasing 3 °C per cycle)	20 s<br>10 s
	Denaturation<br>Annealing/ extension<br>+ Plate read	40	95<br>58	15 s<br>15 s
	Melt curve<br>+ Plate read	1	60-94<br>(1 °C increment)	5 s
igm	Initial denaturation	1	95	3 min
	Denaturation<br>Touchdown stage	4	95<br>66 (by decreasing 3 °C per cycle)	20 s<br>10 s
	Denaturation<br>Annealing/ extension<br>+ Plate read	40	95<br>58	15 s<br>15 s
	Melt curve<br>+ Plate read	1	60-94<br>(1 °C increment)	5 s
mt	Initial denaturation	1	95	3 min
	Denaturation<br>Touchdown stage	4	95<br>66 (by decreasing 3 °C per cycle)	20 s<br>10 s
	Denaturation<br>Annealing/ extension<br>+ Plate read	40	95<br>60	15 s<br>15 s
	Melt curve<br>+ Plate read	1	60-94<br>(1 °C increment)	5 s

mir132	Initial denaturation	1	95	2 min
	Denaturation<br>Annealing/ extension<br>+ Plate read	40	95<br>56	10 s<br>60 s
	Melt curve<br>+ Plate read	1	60-95<br>(1 °C increment)	5 s
mir155	Initial denaturation	1	95	2 min
	Denaturation<br>Annealing/ extension<br>+ Plate read	40	95<br>56	10 s<br>60 s
	Melt curve<br>+ Plate read	1	60-95<br>(1 °C increment)	5 s

Table S3 Summary of Markov chain Monte Carlo (MCMC) models for gene expression differences observed between Pb-exposed and control shorthorn sculpin (*M. scorpius*) and between tank in each treatment.

Gene	Treatment/tank	Fold change[a]	p_{MCMC}[b]
hsp70	Treatment (Pb exposure:control)	-0.10082	0.870
	Tanks (within Pb exposure)	-0.47393	0.320
	Tanks (control)	-0.67679	0.304
IgM	Treatment (Pb exposure:control)	1.52622	0.028 *
	Tanks (Pb exposure)	-0.52371	0.400
	Tanks (control)	1.11949	0.094
mt	Treatment (Pb exposure:control)	1.23757	0.030 *
	Tanks (Pb exposure)	-0.08002	0.784
	Tanks (control)	1.23143	0.062
mir132	Treatment (Pb exposure:control)	0.07169	0.784
	Tanks (Pb exposure)	-0.12861	0.588
	Tanks (control)	0.30919	0.222
mir155	Treatment (Pb exposure:control)	0.21447	0.576
	Tanks (Pb exposure)	0.02493	0.916
	Tanks (control)	-0.10075	0.786

[a] Negative numbers indicate a fold-change in the opposite direction of the given amplitude.

[b] Estimating empirical two-tailed *p*-value generated from the results of MCMC sampling. Grey shading denotes statistically significant differences ($p_{MCMC} < 0.05$). The text contains information on the genes studied as well as the experimental regimes.
